# Strategies of Epstein-Barr virus to evade innate antiviral immunity of its human host

**DOI:** 10.3389/fmicb.2022.955603

**Published:** 2022-07-22

**Authors:** Manuel Albanese, Takanobu Tagawa, Wolfgang Hammerschmidt

**Affiliations:** ^1^Max von Pettenkofer Institute and Gene Center, Virology, National Reference Center for Retroviruses, Faculty of Medicine, Ludwig Maximilian University of Munich, Munich, Germany; ^2^Istituto Nazionale di Genetica Molecolare, “Romeo ed Enrica Invernizzi,” Milan, Italy; ^3^Research Unit Gene Vectors, EBV Vaccine Development Unit, Helmholtz Zentrum München, German Research Center for Environmental Health, Munich, Germany; ^4^German Center for Infection Research (DZIF), Partner Site Munich, Munich, Germany; ^5^HIV and AIDS Malignancy Branch, National Cancer Institute, National Institutes of Health, Bethesda, MD, United States

**Keywords:** herpesvirus, interferon, microRNA, immune evasion, natural killer cell, antiviral response, T cell, B cell

## Abstract

Epstein-Barr virus (EBV) is a double-stranded DNA virus of the Herpesviridae family. This virus preferentially infects human primary B cells and persists in the human B cell compartment for a lifetime. Latent EBV infection can lead to the development of different types of lymphomas as well as carcinomas such as nasopharyngeal and gastric carcinoma in immunocompetent and immunocompromised patients. The early phase of viral infection is crucial for EBV to establish latency, but different viral components are sensed by cellular sensors called pattern recognition receptors (PRRs) as the first line of host defense. The efficacy of innate immunity, in particular the interferon-mediated response, is critical to control viral infection initially and to trigger a broad spectrum of specific adaptive immune responses against EBV later. Despite these restrictions, the virus has developed various strategies to evade the immune reaction of its host and to establish its lifelong latency. In its different phases of infection, EBV expresses up to 44 different viral miRNAs. Some act as viral immunoevasins because they have been shown to counteract innate as well as adaptive immune responses. Similarly, certain virally encoded proteins also control antiviral immunity. In this review, we discuss how the virus governs innate immune responses of its host and exploits them to its advantage.

## Introduction

Epstein-Barr virus (EBV), also referred to as human herpesvirus 4 (HHV-4) is a virus of the Gammaherpesvirinae subfamily with an encapsidated, linear, double-stranded DNA genome of approximately 160 kbs and an envelope of about 180 nm in diameter. More than 95% of the adult population worldwide is infected. Primary infection occurs mainly via saliva and is often asymptomatic in childhood, but is frequently associated with Infectious Mononucleosis (IM), if the first contact is delayed until adolescence or adulthood. IM patients have a higher risk of developing Multiple Sclerosis or malignant diseases later in life (Crawford, 2001; Ascherio and Munger, 2015). After infection, the virus establishes latency in mature B cells and persists in long-lived memory B cells. EBV is associated with certain B cell malignancies such as Hodgkin lymphoma, Burkitt’s lymphoma, and post-transplant lymphoproliferative disorder (PTLD) but the virus also causes nasopharyngeal carcinoma and gastric carcinoma in epithelial cells, which EBV can also infect. In immunocompromised individuals, EBV causes PTLD and the majority of acquired immunodeficiency syndrome (AIDS)-defining non-Hodgkin’s lymphoma (Yarchoan and Uldrick, 2018). EBV is a critical human tumor virus and a class 1 carcinogen (International Agency for Research on Cancer [IARC], 1997).

Epstein-Barr virus supports its lifelong persistence in us by minimizing the expression of viral proteins to limit viral detection and to escape from antiviral immunity. During EBV’s life cycle, three different latent phases are characterized by distinct viral gene expression profiles: latency 0/I [no viral proteins or only EBV nuclear antigen 1 (EBNA1)]; latency III [all latent viral proteins (6 EBNAs, 3 latent member proteins; LMPs)]; and latency II (a subset of latent viral proteins). During latency, up to 44 microRNAs (miRNAs) and two non-coding RNAs (EBERs) are also expressed. In *in vitro* infected human B cells, various lytic viral genes are expressed initially together with latent EBV genes in the so-called pre-latent phase of infection (Kalla et al., 2010; Kalla and Hammerschmidt, 2012), but lytic gene expression ceases when the incoming EBV DNA becomes epigenetically repressed within a few days.

During latent infection in resting cells, several copies of the EBV genome are stably maintained as fully chromatinized extrachromosomal plasmids which, in proliferating cells, replicate in synchrony with host cell DNA. To allow virus to spread to neighboring cells, reactivation occurs in B cells when they encounter their cognate antigen *in vivo* (Laichalk and Thorley-Lawson, 2005), but sporadic entry into EBV’s productive, lytic phase also occurs spontaneously in latently EBV infected cell lines *in vitro*. Lytic reactivation is initiated by the viral protein BZLF1, a member of the activator protein 1 (AP-1) family of transcription factors, which induces a massive synthesis of all viral proteins needed for the assembly and egress of virions (Buschle and Hammerschmidt, 2020).

The innate immune system is the frontline defense to protect the host from viral infection (Li and Wu, 2021). B cells and epithelial cells that EBV infects but also monocytes-derived macrophages (MDMs), dendritic cells (DCs), and plasmacytoid dendritic cells (pDCs) can sense virion components. These immune cells express a plethora of pattern recognition receptors, PRRs. Different PRRs recognize different viral components: Toll-like receptor 2 (TLR2) senses the viral dUTPAse in monocytes (Gaudreault et al., 2007; Ariza et al., 2009); in monocytes and pDCs and TLR9 senses the unmethylated CpG motifs of virion DNA (Fiola et al., 2010; Bouvet et al., 2021). The EBERs are recognized by TLR3, TLR7, and Retinoic acid-inducible gene I (RIG-I) in different types of established cell lines (Samanta et al., 2006; Iwakiri et al., 2009; Li et al., 2019) but not when EBV infects primary B cells (Bouvet et al., 2021), which is an unexpected finding. EBV is sensed by interferon gamma inducible protein 16 (IFI16) (Ansari et al., 2013) in epithelial cells in which EBV induces TRIM29, a negative regulator of STING (Xing et al., 2017) (stimulator of interferon genes) and an adaptor protein of cytosolic DNA sensors. A STING inhibitor suppressed B cell transformation by EBV (Miyagi et al., 2021) although B cells barely express STING (Mrozek-Gorska et al., 2019). Further research is needed to clarify its role in EBV infection.

Despite the known innate immune responses EBV establishes long-term latency in most of us, suggesting that it has evolved to escape from human immune recognition. EBV uses several viral gene products with immune evasive functions in different phases of the viral life cycle since many viral components are antigens and are recognized by the host. We summarize recent advancements in understanding the diverse classes of molecules that EBV uses to evade innate immune responses.

## BPLF1

BPLF1 is a large (3149 aa) EBV protein expressed during EBV’s lytic phase and contained in the tegument of virions. BPLF1 stabilizes RAD18 ubiquitin complexes and acts as a deneddylase (removing the ubiquitin-like protein NEDD8) important for lytic viral DNA replication and production of infectious virions (Gastaldello et al., 2010; Kumar et al., 2014). Infection of humanized mice with a BPLF1-knockout virus showed reduced infectivity and delayed B cell transformation suggesting that BPLF1 contributes to but is not essential for the virus to spread *in vivo* (Whitehurst et al., 2015). The N-terminal region of the protein contains a deubiquitinating (DUB) domain, which interacts with proliferating cell nuclear antigen (PCNA) to reduce the cellular DNA damage response after lytic reactivation (Whitehurst et al., 2012). The DUB domain also reduces immune recognition during lytic activation by removing polyubiquitin chains from various components of TLR signaling, including NEMO, IKKα, and TRAF6 (Saito et al., 2013; van Gent et al., 2014). BPLF1 was further reported to interact with the 14-3-3 protein and the E3 ligase tripartite motif-containing protein 25 (TRIM25) mitigating type I interferon (IFN)-mediated responses by sequestering TRIM25 into a trimeric complex. Since RIG-I’s functions depend on ubiquitination by TRIM25, its inhibition causes the loss of the ubiquitin scaffold on RIG-I and affects successive downstream signaling cascades (Gupta et al., 2019). The decreased NF-κB activation in response to EBV reactivation reduces pro-inflammatory cytokine secretion levels. BPLF1 may also have an immune suppressive role immediately after B cell infection since BPLF1 contained in EBV’s infectious particles can control TLR signaling (van Gent et al., 2014) and BPLF1 is also transiently expressed during EBV’s pre-latent phase (Mrozek-Gorska et al., 2019).

## EBV nuclear antigen 1

EBV nuclear antigen 1 is a homo-dimeric protein expressed in all types of viral latency but latency 0. EBNA1 has crucial functions to maintain EBV genomes stably, to replicate viral DNA, and to partition the newly replicated EBV genomes to daughter cells. EBNA1 binds to the EBV origin of plasmid replication (*oriP*), which is critical for maintaining and replicating the viral genome and for its partitioning during mitosis (Chiu and Sugden, 2018). EBNA1’s amino-terminus binds cellular chromatin to localize EBV genomes to perichromatin and contains a glycine alanine repeat (GAr) domain with immune evasive function reducing presentation of EBNA1 epitopes to CD8^+^ cytotoxic T cells (Blake et al., 1997; Yin et al., 2003).

EBNA1’s role in regulating innate immunity is less clear. It interferes with functional NF-κB activation in different carcinoma cell lines (Valentine et al., 2010) and it can contribute to the development of EBV-associated tumors and their progression by activating AP-1 and signal transducer and activator of transcription 1 (STAT1) (O’Neil et al., 2008) and inhibiting TGF-β1 (Wood et al., 2007; Flavell et al., 2008). This is counterintuitive because STAT1 activation typically induces strong anti-viral IFN responses. EBV suppresses these “adverse effects” together with other immunoevasins such as EBNA2 or viral miRNAs. EBNA1 also induces CXCL12 secretion to recruit regulatory T cells and to create an immunosuppressive microenvironment (Huo et al., 2020). Finally, EBNA1 also regulates the expression of NKG2D ligands which reduces the recognition of EBV infected cells by NK cells shortly after infection (Westhoff Smith et al., 2021).

## EBNA2

EBNA2 acts as the main transcriptional transactivator (Kempkes and Ling, 2015) and is essential for the reprogramming and transformation of primary B cells infected by EBV *in vitro* (Hammerschmidt and Sugden, 1989). EBNA2 induces a plethora of cellular genes including *MYC* (Kaiser et al., 1999) and genes coding for cell surface and adhesion molecules such as CD21 and CD23, but EBNA2 also activates multiple viral proteins including EBNA1, LMP1, and LMP2 (Kempkes and Ling, 2015). Regarding EBNA2’s role in innate immunity, it induces the expression of the IL-18 receptor (IL-18R) in primary B cells (Pagès et al., 2005). IL-18R may function differently depending on its ligands, the pro-inflammatory and anti-inflammatory cytokines IL-18 and IL-37, respectively. With IL-37, IL-18R can inhibit innate immune responses in general (Nold-Petry et al., 2015) but the contribution of the IL-18R pathway to EBV’s strategy of immune evasion needs to be assessed. EBNA2 was also shown to upregulate TNF-α and LT-α to support cell proliferation (Spender et al., 2001). EBNA2 also regulates host miRNAs hsa-miR-21 and miR-146a that act as negative feedback of type I IFN responses in the RIG-I pathway (Rosato et al., 2012). Already in the 1990s, EBNA2 was described to render the infected cells insensitive to IFN (Aman and von Gabain, 1990) and to suppress interferon stimulated genes (ISGs) but EBNA2 also induces low levels of IFN-β suggesting that chronic low levels of IFN may desensitize cells and dampen proper anti-viral responses (Kanda et al., 1992, 1999). In 293T and HeLa cells, EBNA2 activates STAT3, which could provide a growth advantage to EBV infected cells (Muromoto et al., 2009).

## BCRF1 (vIL-10)

Epstein-Barr virus also manipulates innate immunity using viral IL-10 (vIL-10) encoded by the BCRF1 gene of EBV, a viral homolog of human IL-10 (Sin and Dittmer, 2012). vIL-10 is an anti-inflammatory cytokine that suppresses secretion of pro-inflammatory cytokines such as IFN-γ and IL-2 from EBV infected PBMCs (Jochum et al., 2012a). NK cells can sense and lyse EBV infected cells particularly during EBV’s lytic phase. CD4^+^ T cells can enhance NK cell activity, but vIL-10 suppresses this adjuvant T cell effect reducing the cytotoxic activity of NK cells (Jochum et al., 2012a). vIL-10 also controls adaptive immunity by suppressing TAP1, class I HLA, and co-stimulatory molecules such as ICAM-1 (Zeidler et al., 1997; Salek-Ardakani et al., 2002). Unlike hIL-10, vIL-10 does not induce class II MHC (Go et al., 1990) or promotes thymocyte proliferation (MacNeil et al., 1990) probably due to its lower receptor affinity compared to hIL-10 (Liu et al., 1997). In a recent study, vIL-10 reduced phosphorylation of STAT3 and vIL-10-treated monocytes showed weaker phagocytosis of apoptotic cells compared to hIL-10-treated cells (Jog et al., 2018). The impaired activity of monocytes to clear dying cells may lead to reduced antigen presentation indirectly supporting EBV infection *in vivo*. vIL-10 is not only a lytic gene but is markedly expressed in the pre-latent phase of B cell infection (Jochum et al., 2012a; Sin and Dittmer, 2012). Mice infected with a strain of murine gammaherpesvirus 76 engineered to encode EBV’s vIL-10 showed higher virus titers in the lung compared to wild-type virus but vIL-10 had no effect on virus reactivation or the number of latently infected splenic cells (Lindquester et al., 2014). Thus, vIL-10 can also contribute to a pro-viral microenvironment early after infection in this mouse model.

## Latent membrane protein 1

Latent membrane protein 1 contains 6 transmembrane domains and a C-terminal signaling domain and is located mainly in intracellular membranes (Lam and Sugden, 2003). LMP1 mimics a constitutive, i.e., ligand-independent CD40 receptor and its downstream signaling cascade (Kilger et al., 1998; Kieser and Sterz, 2015) contributing to survival and proliferation of EBV infected B cells (Wang et al., 1985; Zimber-Strobl et al., 1996; Kilger et al., 1998). LMP1 drives an innate anti-viral response but it also evokes an anti-viral state in infected cells. LMP1 induces interferon regulatory factor 7 (IRF7) via TRAF6 and RIP mediated ubiquitination (Zhang and Pagano, 2000; Huye et al., 2007; Ning et al., 2008) and activates the JAK/STAT pathway as well as both canonical and non-canonical NF-κB signaling pathways (Gires et al., 1999; Zhang L. et al., 2004; Huye et al., 2007). As a consequence, EBV infected cells release type I and II interferons leading to an upregulation of ISGs including ISG15, STAT1, and 2′,5′-oligoadenylate synthetase (OAS) (Richardson et al., 2003; Zhang J. et al., 2004; Najjar et al., 2005). Using LMP1 mutants, STAT1 regulation was shown to depend on both signaling domains of LMP1, CTAR1, and CTAR2 (Zhang L. et al., 2004). The authors suggested that this anti-viral response can be advantageous for the virus because it also supported latent infection in their experimental model (Zhang J. et al., 2004; Zhang L. et al., 2004). Of note, most of these reports rely on latently EBV infected, established B cell lines.

Recent reports identified additional immune evasive functions of LMP1. LMP1 downregulated *TLR9* and reduced the recognition of non-CpG methylated viral DNA (Fathallah et al., 2010). Later, newly EBV infected B cells were found to be rather inert to sensing viral DNA (Bouvet et al., 2021), but plasmacytoid dendritic cells (pDCs) released high levels of type I IFN upon encounter with viral DNA and its detection by TLR9 (Bouvet et al., 2021). In 293T cells, LMP1 expression promoted RIG-I degradation (Xu et al., 2018), which might also dampen antiviral responses.

## Latent membrane protein 2

The viral LMP2 gene encodes two proteins, LMP2A and LMP2B, which differ in their N-terminal domains. Both LMP2A and LMP2B are integral transmembrane proteins embedded in the plasma membrane with their 12 transmembrane spanning domains. LMP2A’s N-terminal domain is cytosolic and contains two proline-rich PY domains as well as an immunoreceptor tyrosine-based activation motif (ITAM). The ITAM motif interacts with signal mediators of the B-cell receptor (BCR), mimics an active BCR (Engels et al., 2001a,b) and maintains a “tonic” B cell receptor-like signal (Mancao et al., 2005) and references therein. Concomitantly, LMP2A contributes to B cell survival as its expression supports viral latency and prevents acute BCR triggering (Brielmeier et al., 1996; Merchant et al., 2000; Mancao and Hammerschmidt, 2007). LMP2B has a short, 19 amino acid long N-terminal domain, only, which is incapable of signaling and was shown to form heterocomplexes with LMP2A regulating LMP2A’s activity (Lynch et al., 2002). LMP2A also controls adaptive immunity by downregulating CIITA, the master regulator of HLA class II alleles, reducing epitope presentation via HLA class II (Lin et al., 2015). LMP2A not only reduces immune recognition by CD8^+^ T cells (Rancan et al., 2015) but also innate immune responses in epithelial cell lines. IL-6 expression and secretion from carcinoma cell lines was suppressed by wild-type EBV but not by an LMP2A-deleted mutant EBV indicating that LMP2A shuts off this inflammatory cytokine (Stewart et al., 2004). LMP2A also downregulated LMP1, suggesting a fine-tuning of LMP1’s activities in EBV infected cells (Stewart et al., 2004) probably mediated by TRAF2 (Guasparri et al., 2008). Because LMP2A can induce the degradation of the receptors IFNAR1 and IFNGR1, LMP2A affects expression of ISGs and thus reduces type I and type II interferon responses in epithelial cells (Shah et al., 2009). Interestingly, LMP2B was also found to contribute to IFN receptor degradation suggesting that the N-terminal cytosolic tail of LMP2A, which LMP2B lacks is not important for this ubiquitin-dependent mechanism (Shah et al., 2009).

## Epstein-Barr virus microRNAs

Epstein-Barr virus encodes 44 mature miRNAs which are important for cell survival and proliferation in the pre-latent phase of B cell infection (Seto et al., 2010; Feederle et al., 2011). Recently, many viral miRNAs were also found to govern innate and adaptive immune responses *in vitro* (Albanese et al., 2016; Tagawa et al., 2016; Bouvet et al., 2021) and in humanized mice *in vivo* (Murer et al., 2019). miRNAs regulate important proteins in different pathways of innate immunity. EBV miR-BART15 targets the NLRP3 (NOD-, LRR-, and pyrin domain-containing protein 3) inflammasome and EBV mir-BHRF1-2 was shown to regulate the IL-1 receptor preventing IL-1β release and downstream activation (Haneklaus et al., 2012; Skinner et al., 2017). EBV miR-BART2 targets *MICB* facilitating immune escape from Natural Killer (NK) cells (Nachmani et al., 2009). EBV miRNAs in primary B cells target key components of the interferon pathway reducing production and release of IFN from infected cells as well as autocrine and paracrine IFN mediated responses affecting ISG-driven transcription (Bouvet et al., 2021). Additional targets involved in reducing type I IFN response are CBP (CREB-binding protein) (Hooykaas et al., 2017) and RIG-I (Lu et al., 2017). Interestingly, pDCs, although they are only abortively infected, sense EBV’s genomic DNA in a TLR9 dependent manner but EBV’s miRNAs seem to counteract this innate response. The mode of action is not completely clear but could be mediated by miRNAs contained in virions (Jochum et al., 2012b; Bouvet et al., 2021). miRNAs are particularly interesting as immunoevasins. Viral proteins including those with anti-inflammatory functions are immunogenic and thus can stimulate adaptive immunity but viral miRNAs escape from immune recognition entirely.

## Epstein-Barr virus lytic proteins

In latently infected cells certain viral genes can control innate immunity, but lytic genes also undermine immune functions. IRF7 was shown to be regulated by the immediate early genes BZLF1 (Hahn et al., 2005) and BRLF1 (Bentz et al., 2010). Similarly, several lytic proteins, BGLF4 (Wang et al., 2009), BRLF1 (Bentz et al., 2010), BHRF1 (Vilmen et al., 2021), and BFRF1 (Wang et al., 2020) target IRF3. It and IRF7 are critical for type I IFN production and downstream ISG signaling, suggesting that the orchestrated suppression of both IRF3 and IRF7 is important in EBV’s lytic, productive phase. BGLF2 was identified to control all three types of IFN (α, β, ε) as it interferes with STAT1/STAT2 (Jangra et al., 2021) and tyrosine kinase 2 (TYK2) functions (Liu et al., 2020). Viral lytic genes also target PRRs. The BGLF5 exonuclease downregulates TLR2 (van Gent et al., 2015) and the dsDNA sensor TLR9 (van Gent et al., 2011). BLRF1 not only inhibits IRF3 but also suppresses transcription of RNA polymerase III-dependent immunogenic small RNAs dampening the RIG-I pathway (Long et al., 2021). Secreted BARF1 is a decoy receptor of macrophage colony stimulating factor (M-CSF) interfering with macrophage activation (Hoebe et al., 2012). Immune evasion appears to be particularly important during latent infection as compared to EBV’s lytic phase, when viral host shut-off mechanisms compromise cellular protein synthesis and thus immune functions (Rowe et al., 2007; Buschle et al., 2021). But, as in the case of BPLF1, viral genes that have been previously deemed lytic genes are now found to be expressed during EBV’s pre-latent phase of infection as well. These lytic immunoevasins are, therefore, potentially functional upon *de novo* infection.

## Discussion

**FIGURE 1:**
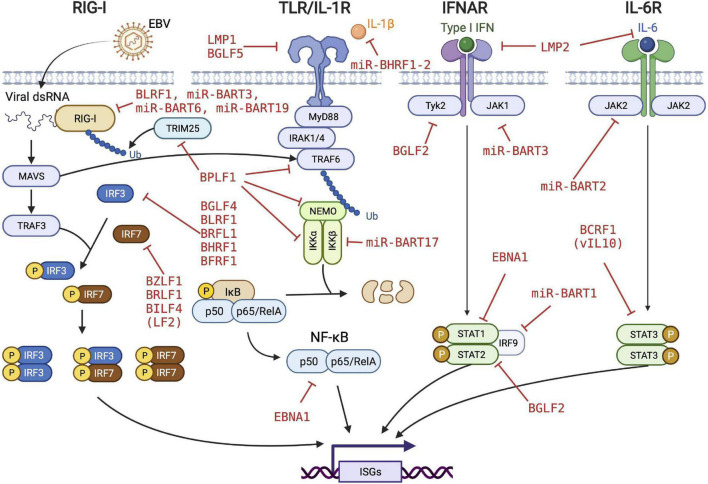
EBV’s strategy to evade innate immune responses. EBV evolved to encode many viral proteins and microRNAs to fend off different levels of innate immune responses of the infected cells. Viral immunoevasins and their regulatory functions are depicted in red. RIG-I, retinoic acid-inducible gene I; TLR, toll-like receptor; IFNAR, interferon-α/β receptor; MAVS, mitochondrial antiviral signaling protein; TRAF, TNF receptor associated factor; IRF, interferon regulatory factor; TRIM, tripartite motif containing; IRAK, interleukin receptor associated kinase; NEMO, nuclear factor-kappa B essential modulator; JAK, janus kinase; STAT, signal transducer and activator of transcription protein; ISG, interferon-stimulated gene. We acknowledge Biorender.com for graphical elements we used to compose the figure.

The data discussed above and summarized in Table 1 and Figure 1 show that EBV has evolved to encode different classes of immune evasins, which contribute to successful infection and stable latency in its human host. Many factors have been found to play various but likely crucial roles in evading innate immunity and probably more factors and new principles will be discovered. Moreover, it appears difficult to assess the importance and contribution of each individual immunoevasin detected or claimed in the literature. Few findings have been independently confirmed or falsified. This uncertainty is partly due to the many different experimental systems and models used by different groups working in this field of virology and antiviral immunity.

**TABLE 1:** Overview of cellular functions regulated by EBV proteins and miRNAs.

	Target	Function	Viral regulator	References
Pattern recognition receptors (PRR)				
	TLR2	Extracellular PRR	BGLF5,	van Gent et al., 2015
	TLR9	Endosomal PRR	LMP1, BGLF5,	Fathallah et al., 2010; van Gent et al., 2011
	RIG-I	Cytosolic PRR	miR-BART6 and miR-BART19, LMP1, miR-BART3, BLRF1	Bouvet et al., 2021; Xu et al., 2018; Lu et al., 2017; Long et al., 2021
	NLRP3	Cytosolic PRR	miR-BART15	Haneklaus et al., 2012
	TRIM25	Adapter (Ubiquitin ligase)	BPLF1	Gupta et al., 2019
	TRAF6	Adapter (Ubiquitin ligase)	BPLF1, LMP1	Saito et al., 2013; van Gent et al., 2014; Huye et al., 2007; Ning et al., 2008; Zhang and Pagano, 2000
	IRAK2	Protein kinase	miR-BART22	Bouvet et al., 2021
	NEMO	Protein kinase	BPLF1	van Gent et al., 2014
	IKKα	Protein kinase	BPLF1	van Gent et al., 2014
	IKKβ	Protein kinase	miR-BART17	Bouvet et al., 2021
	NF-κB	Transcription factor	EBNA1	Valentine et al., 2010
	IRF3	Transcription factor	BRLF1, BGLF4, BHRF1, BFRF1	Bentz et al., 2010; Wang et al., 2009; Vilmen et al., 2021; Wang et al., 2020
	IRF7	Transcription factor	BZLF1, BRLF1, BILF4 (LF2)	Hahn et al., 2005; Bentz et al., 2010; Wu et al., 2009
IFN pathway				
	IFNAR1	Extracellular receptor	LMP2	Shah et al., 2009
	IFNAR2	Extracellular receptor	LMP2	Shah et al., 2009
	JAK1	Protein kinase	miR-BART3	Bouvet et al., 2021
	JAK2	Protein kinase	miR-BART2	Bouvet et al., 2021
	TYK2	Protein kinase	BGLF2	Liu et al., 2020
	STAT1	Transcription factor	EBNA1	O’Neil et al., 2008; Wood et al., 2007
	STAT2	Transcription factor	BGLF2	Jangra et al., 2021
	STAT3	Transcription factor	EBNA2, BCRF1 (vIL-10)	Muromoto et al., 2009; Jog et al., 2018
	IRF9	Transcription factor	miR-BART1	Bouvet et al., 2021
Cytokines				
	IL-6	Cytokine	LMP2	Stewart et al., 2004
	M-CSF	Cytokine	BARF1	Hoebe et al., 2012
	TGFβ1	Cytokine	EBNA1	Flavell et al., 2008; Wood et al., 2007; Huo et al., 2020
	IFN-γ	Cytokine	BCRF1 (vIL-10)	Jochum et al., 2012a
	IL-2	Cytokine	BCRF1 (vIL-10)	Jochum et al., 2012a
	TNF-α	Cytokine	EBNA2	Spender et al., 2001
	LT-α	Cytokine	EBNA2	Spender et al., 2001
	IFN-β	Cytokine	EBNA2	Kanda et al., 1992; Kanda et al., 1999
	IL-1R	Receptor	miR-BHRF1-2	Skinner et al., 2017
	TRAF2	Signal transducer	LMP2	Guasparri et al., 2008
Gene regulation				
	CBP	Transcription factor	miR-BART16	Hooykaas et al., 2017
	PCNA	Replication factor	BPLF1	Whitehurst et al., 2015; Whitehurst et al., 2012
	hsa-miR-21	microRNA	EBNA2	Rosato et al., 2012
	hsa-miR-146a	microRNA	EBNA2	Rosato et al., 2012
NK cell function				
	NKG2D	Activating receptor	EBNA1	Westhoff Smith et al., 2021
	MICB	Receptor	miR-BART-2	Nachmani et al., 2009

Use of models that closely mimic *in vivo* infection is a potential solution to this problem. We recently showed that primary human B cells constitute a valid model to assess the importance of candidates for immune evasion in the different phases of EBV infection. Using genetic EBV mutants that differ only in single genes are also supportive. For example, we found that the EBV gene BILF4 (LF2), which was shown to play a role in restricting innate immune response (Wu et al., 2009), did not show any reduction in IFN response in freshly infected B cells (Bouvet et al., 2021). This result suggested that data obtained from phenotypic studies in established cell lines are not necessarily relevant in models recapitulating critical steps in infection of EBV’s genuine target cells. A better understanding of the virus and its crucial factors that are responsible for successful infection will be important to identify potential therapeutic targets to prevent or cure EBV-associated diseases.

## Author contributions

MA, TT, and WH conceptualized the topic. MA and TT researched and analyzed the literature, and drafted the manuscript including interpretations. WH analyzed the background literature, and wrote and finalized the manuscript together with MA and TT. All authors approved the final version of the manuscript, ensured the accuracy and integrity of the work, and agreed to be accountable for all aspects of the work.

## Conflict of interest

The authors declare that the research was conducted in the absence of any commercial or financial relationships that could be construed as a potential conflict of interest.

## Publisher’s note

All claims expressed in this article are solely those of the authors and do not necessarily represent those of their affiliated organizations, or those of the publisher, the editors and the reviewers. Any product that may be evaluated in this article, or claim that may be made by its manufacturer, is not guaranteed or endorsed by the publisher.
